# Healthy Food Procurement Policies and Their Impact

**DOI:** 10.3390/ijerph110302608

**Published:** 2014-03-03

**Authors:** Mark L. Niebylski, Tammy Lu, Norm R. C. Campbell, Joanne Arcand, Alyssa Schermel, Diane Hua, Karen E. Yeates, Sheldon W. Tobe, Patrick A. Twohig, Mary R. L’Abbé, Peter P. Liu

**Affiliations:** 1 Libin Cardiovascular Institute of Alberta, University of Calgary, 3280 Hospital Drive NW, Calgary, AB T2N 4Z6, Canada; E-Mails: mniebylski@yahoo.com (M.L.N.); tamlu@ucalgary.ca (T.L.); 2 Department of Nutritional Sciences, Faculty of Medicine, University of Toronto, 150 College St., Toronto, ON M5S3E2, Canada; E-Mails: joanne.arcand@utoronto.ca (J.A.); a.schermel@gmail.com (A.S.); mary.labbe@utoronto.ca (M.R.L.’A.); 3 Sunnybrook Health Sciences Centre, Sunnybrook Research Institute, University of TorontoBayview Ave. E239, Toronto, ON M4N 3M5, Canada; E-Mails: Diane.Hua@sunnybrook.ca (D.H.); sheldon.tobe@sunnybrook.ca (S.W.T.); 4 Department of Medicine, Queen’s University, 2059 Etherington Hall, Kingston, ON K7L 3N6, Canada; E-Mail: yeatesk@queensu.ca; 5 Toronto General Hospital, University of Toronto, 200 Elizabeth St., Toronto, ON M5G 2C4, Canada; E-Mails: ptwohig@uhnresearch.ca (P.A.T.); peter.liu@utoronto.ca (P.P.L.)

**Keywords:** public policy, health promotion, health, food, non-communicable disease, sodium, sugar, saturated fat, trans fatty acids

## Abstract

Unhealthy eating is the leading risk for death and disability globally. As a result, the World Health Organization (WHO) has called for population health interventions. One of the proposed interventions is to ensure healthy foods are available by implementing healthy food procurement policies. The objective of this systematic review was to evaluate the evidence base assessing the impact of such policies. A comprehensive review was conducted by searching PubMed and Medline for policies that had been implemented and evaluated the impact of food purchases, food consumption, and behaviors towards healthy foods. Thirty-four studies were identified and found to be effective at increasing the availability and purchases of healthy food and decreasing purchases of unhealthy food. Most policies also had other components such as education, price reductions, and health interventions. The multiple gaps in research identified by this review suggest that additional research and ongoing evaluation of food procurement programs is required. Implementation of healthy food procurement policies in schools, worksites, hospitals, care homes, correctional facilities, government institutions, and remote communities increase markers of healthy eating. Prior or simultaneous implementation of ancillary education about healthy eating, and rationale for the policy may be critical success factors and additional research is needed.

## 1. Introduction

A growing proportion of the global population has diet-related non-communicable health risks and diseases (NCDs), such as obesity, hypertension, dyslipidemia, diabetes, heart disease, stroke, or cancer [1,2,3,4,5,6,7,8,9,10,11,12,13,14,15,16,17]. These largely result from unhealthy lifestyle choices in unhealthy living environments, and cost billions of dollars every year, threatening economies and the sustainability of health care systems around the world [1,18]. NCDs account for over 63% of deaths and it is estimated that 40% of these NCD-related deaths are attributed to diet [19,20,21,22,23]. The main dietary factors causing disease are excess intakes of free sugar, saturated fats and *trans*-fatty acids, and sodium, much of which is added during food processing and a lack of fruits and vegetables [1,2,3,4,5,6,7,24]. To reduce the burden of NCDs, there is a subsequent call for population health interventions to improve the quality of dietary intakes [18]. 

There are several potential policy interventions that can support healthy eating. Healthy food procurement policies require that the food purchased, provided, or made available is healthy (or at least healthier) and the policies are often directed at people who have a large proportion of their daily intake from a central organization (e.g., schools) [25,26,27]. A definition of healthy food procurement that has been used in a review of policies is “a process which encompasses not just how public bodies procure food, but also how they determine what food they want to buy and from whom; receive and store food; prepare and serve food; dispose of waste food; and monitor their costs” [26]. Broad implementation of healthy food procurement policies have the potential to increase the overall demand for more healthy products, drive the reformulation of foods by food manufacturers, and increase the availability of healthier foods to the general public [25,26]. Procurement policies have been indicated to be relatively inexpensive to implement, can encourage local production of foods if the policy requires sourcing food from local growers, and raise awareness about the importance of a healthy diet if coupled with education [26]. However, despite the potential for healthy food procurement interventions, they have not been broadly implemented, perhaps in part because of a lack of clear understanding of the impact of the policies that have been implemented. We conducted this review to identify healthy food procurement policies that have been evaluated for their impact on healthy eating and health outcomes.

## 2. Experimental Section

A comprehensive search strategy was developed to identify articles that assessed the impact of healthy food procurement interventions. The databases PubMed (1964‒27 July 2012), and Medline (1950‒27 July 2012) were searched using the terms: “food procurement”, “procurement policy”, “procurement intervention”, “food procurement policy”, “healthy food catering”, “nutrition standards”, “food procurement intervention” and “healthy food policy”. Three reviewers examined titles and abstracts for randomized controlled trials and prospective and retrospective non randomized food procurement interventions that assessed the impact on: (1) nutrition related health indicators to include blood pressure, body mass index (BMI), body weight, blood lipids or glucose, (2) healthy food purchases by consumers, (3) consumption of healthier foods or (4) knowledge, attitudes or behaviors towards healthy foods. Full text articles were obtained and those that were not in English, did not involve humans, were based on data previously published, or were not full reports (*i.e.*, abstracts) were excluded. The studies were classified into the primary site of the intervention (school, worksite, hospital, care home, correctional facility, government institution and remote community).

In addition, Google Scholar (July 2012) was searched and individuals at the World Health Organization (WHO), Pan American Health Organization (PAHO), Department of Health–—Nutrition Branch in England, Centers for Disease Control and Prevention (CDC) in the USA, New York City Department of Health and Mental Hygiene*,* Heart Foundation in Australia, Government of New Zealand, and the Public Health Agency of Canada were contacted to determine if there were government interventions that may not have been published. These “grey” literature documents included government publications, recently completed studies, or unpublished materials. The references of publications were searched for additional relevant citations. 

## 3. Results and Discussion

**Figure 1:**
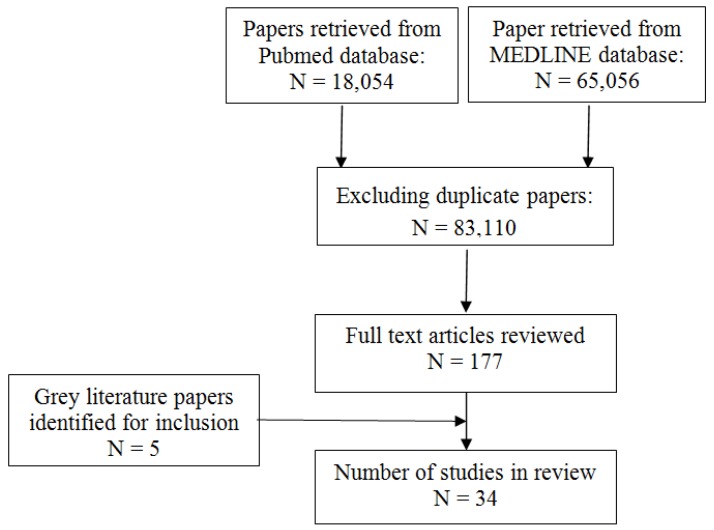
Selection of articles for review *.

Notes: * Studies that were included if they were: (1) randomized controlled trials, prospective and retrospective non randomized food procurement interventions that assessed the impact on: (a) nutrition related health indicators to include blood pressure, body mass index (BMI), body weight, blood lipids or glucose, (b) healthy food purchases by consumers, (c) consumption of healthier foods or (d) knowledge, attitudes or behaviors towards healthy foods, and (2) full text articles, written in English. Studies that were duplicate publications and did not involve humans were excluded.

The PubMed database search retrieved 18,054 citations references while the Medline search retrieved 65,056 citations (Figure 1). The searches identified 83,110 citations when duplicate citations were excluded. One hundred and seventy seven full articles were reviewed, and, of these, 34 were found to meet the inclusion criteria of this review. The selected articles were placed into intervention categories based on setting as detailed below.

### 3.1. Interventions in Schools

Multiple healthy food policies for schools have been developed (Table 1). In 2008, England introduced a national regulation that requires all primary schools to use a healthy food procurement standard for foods throughout the school day [28,29,30]. These regulations impacted 136 primary schools and improved the purchases of fruits, vegetables, and salads by 15%, and reduced processed foods high in sodium, fats, and sugars by 12% (e.g., French fries, pizza, and cookies) [28]. Following implementation, 74% of students indicated a greater desire for healthier foods, and there was a 15% increase in the purchase of healthier foods in cafeterias from 2006 to 2009 (Table 2) [28]. These improvements may also be attributed in part to concurrent educational programs that emphasized the importance of a healthy diet. In 2011, the Department of Education implemented a similar healthy food program in English secondary schools (Table 2) [31]. Dietary intake data was collected for 6,000 secondary schools students from 79 schools. The food procurement intervention reduced the sodium (18%), sugar (4%), and fat (5%) content of several foods served in the participating schools. Analysis of dietary intake among students found a 16% reduction in energy intake, 27% reduction in fat, 18% reduction in sodium, and 37% reduction in sugar intake (Table 1) [32].

“The Fresh Program” in California, USA encouraged the growth and use of local foods rather than processed foods, provided funding opportunities to small and medium sized farms, and educated students about the importance of a healthy diet [33]. The “Fresh Program” resulted in a 58% increase in fruit and vegetable sales, and 65% of students selected healthier menu items over foods high in fat, sugar and sodium (Table 1) [33]. 

In 2005, British Columbia Canada, introduced *Guidelines for Food and Beverage Sales in BC Schools*, which has led to 50% of schools eliminating foods that are “not recommended” by this program (e.g., soups with >750 mg of sodium per serving) [34]. A similar evaluation performed in 2007 found that schools who had yet to totally eliminate “not recommended” foods had reduced them under 20% of the total food sold in school vending machines and cafeterias [34]. California implemented a school-based program, and found that approximately 67% of schools were compliant with state standards, but no evaluation of changes in food intake pre- and post-intervention was performed [35,36,37].

There have been additional evaluations of healthy food procurement interventions in school settings using different methodologies(Table 1) [26,27,28,31,33,38,39,40,41,42,43,44,45,46,47,48,49,50,51,52]. Each of these studies had variations in sample size, age of students, duration, and educational component, and one included an intervention to promote physical activity. Despite these variations, all the food procurement interventions in school settings demonstrated increases in healthy food purchasing patterns (Table 1).

Many of the school interventions that also included an education component were effective at increasing the intake of healthy foods and decreasing the intake of foods high in fat, sodium, and sugar. Two studies that assessed health outcomes found a reduction in blood pressure and BMI [39,42]. In these studies, procurement of food involved providing greater quantities and lowering the price of healthy foods in cafeterias and vending machines. The studies were implemented without any perceived barriers.

**Table 1:** Healthy Food Procurement Programs in Schools.

Study and Year	Study Description	Intervention(s)	Post-intervention Outcome(s) and Notable Findings
School Food Trust 2009 [28]	At 136 primary schools in England, dietary intake was assessed and compared with 2005 survey results.	Increased provision of healthy foods and decreased the availability of foods high in sodium, fat, and sugar.	Consistent benefits from 2005 to 2009 and included decreased sugar, fat, and saturated fat intake. Further, these lunches in 2009 contained almost one-third less sodium compared with 2005.
School Food Trust 2011 [31]	At 80 secondary schools in England, dietary intake was assessed and compared with 2004 results [32].	Increase provision of healthy foods and decreased the availability of unhealthy foods high in sodium, fat, and sugar.	The average meal contained >30% less saturated fat, total fat, sodium and sugar and 50% more vitamin A than in 2004 and there was a 6% increase in F&V intake since then.
School Food Trust 2004 [32]	At 79 secondary schools in England, dietary intake was assessed.	Improved the nutritional quality of foods served in the schools and performed surveys.	On average, the intervention reduced dietary total fat, saturated fat, sugar, sodium, and energy intake by 27%, 23%, 37%, 18%, and 16%, respectively, in the schools. Survey results were with 2011 results [31].
Joshi *et al.* 2005 [33]	This report showcases innovative farm to school programs from around the USA to include eight case studies.	Predominantly provision of fresh foods from local farms along with education though innovative strategies are detailed.	Results varied with each state’s intervention(s). In California, 65% of students chose healthier menu items over meals high in fat, sugar and sodium and increased intake of F&V by 58%. There were an estimated 950 “Fresh” programs in the USA by 2006.
Simons-Morton *et al.* 1991 [38]	Four elementary schools for K-4th grade students in Texas (USA) with two being controls evaluated the impact of a school-based program on improving diet and physical activity.	The three intervention components were classroom education (Go For Health Curriculum), physical activity (Children’s Active Physical Education), and low fat/low sodium school lunches (New School Lunch).	The two intervention schools had decreased total fat (15.5%; 10.4%), saturated fat (31.7%; 18.8%), and sodium (40.3%; 53.6%). Physical activity increased from less than 10% class time to 40% of class time. Adoption of such programs in other schools may be a challenge.
Ellison *et al.* 1989 [39]	Food service workers ate two high schools in Massachusetts and New Hampshire modified preparation of foods served at dining halls.	Increased availability of healthier food through the food service providers and assessed in all students taking a science course.	Reduced sodium intake by 15%–20% and saturated fat intake by 20%. The lower sodium intake over a school year resulted in lower blood pressure among students receiving the intervention. Intervention was well received by workers and students.
Jeffrey *et al.* 1994 [40]	Cafeteria at a university office building housing 700 employees in Minnesota (USA).	Increased availability and reduced price of fruit and salads in a school cafeteria and assessed by daily sales.	Three-fold increase in the sale of fruit and salad after reducing the price by 50% over a 3-wk period. Women were more prone to make more nutritious purchases.
French *et al.* 2001 [41] (Also in Table 2)	Examined the impact of pricing and promotion of low-fat snacks in vending machines at 12 worksites and 12 schools in Minnesota (USA) over a 12-month period.	Low-fat snacks added to 55 vending machines were subject to four pricing conditions and three promotional conditions and sales were tracked.	Price reductions of 10%, 25%, and 50% were associated with significant increases in sales of low-fat snacks in adults and adolescents. Profits per vending machine were not impacted and promotional signage may have had slight effect.
Saksvig *et al.* 2005 [42]	Ojibway-Cree First Nations 3rd, 4th and 5th grades students with school-based program delivered at the Sandy Lake School in northern Canada assessed at baseline and one year later at follow-up.	Culturally appropriate diabetes prevention program that banned high-fat and high-sugar snack foods and provided a healthier lunch. Included education on diet and physical activity with community support.	This program was associated with improved dietary knowledge, dietary self-efficacy, and understanding of the psychosocial factors related to healthy eating and dietary fiber intake of students in a remote First Nations community. The intake of energy from total fat decreased significantly by 2% after one year.
Auld *et al.* 1998 [43]	Comprehensive, 4-yr program in three Denver, CO (USA) elementary schools aimed at increasing consumption of whole grains, F&V with nutrition education.	Integrated Nutrition Project included 24 weekly hands-on, teacher led activities; six parent-taught lunchroom mini-lesson.	Students in treatment classrooms achieved significantly greater gains in knowledge and self-efficacy on food preparation and F&V consumption. Integrated approach with education and healthy food procurement may increase desire for healthier foods.
French *et al.* 2004 [44]	20 secondary schools in Minnesota participated over two years with a portion serving as controls.	Environmental intervention in school cafeterias where they increased availability of lower fat foods and implemented student-based promotions.	There was a significant increase (35%) in the sales of lower-fat foods in the intervention group and a significant increase in lower-fat foods in the al a carte product mix.
Perry *et al.* 1998 [45]	20 primary schools in Minnesota used a multi-component approach to increase F&V consumption in 4th/5th grade students over a 2 year period.	The 5-a-Day Power Plus Program included behavioral curricula in the classroom, parental involvement, school food changes, and industry involvement.	The program significantly increased lunchtime F&V consumption; fruit consumption; vegetable consumption among girls.
Perry *et al.* 2004 [46]	The project was performed at 26 elementary schools (grades 1–4) in Minnesota (USA) over two consecutive school years.	The Cafeteria Power Plus project sought to increase the daily availability, attractiveness, and encouragement for F&V with kick-offs, samplings, and challenge weeks. Training of food-service staff and cook managers was ongoing.	Students in the intervention schools significantly increased their total fruit intake. Process measures indicated that verbal encouragement by food-service staff was associated with outcomes. The outcomes suggest that multicomponent interventions are more powerful than cafeteria programs alone with this age group.
Lytle *et al.* 2006 [47]	As part of the TEENS study, 16 middle schools in Minnesota (USA) with approximately 3,600 students in the eight intervention schools were exposed to a multi-component intervention from 1997–2000.	The TEENS intervention included classroom-based curricula, family newsletters, and changes in the school food environment including increasing more healthful options on a la carte and on the school lunch line top increase the availability of F&V and lower fat foods in homes and schools.	Parents of students in intervention schools reported making significantly more healthy food choices when shopping than parents of students in control schools. Compared to control schools, intervention schools offered (*p* = 0.04) and sold (*p* = 0.07) a significantly higher proportion of healthier foods on a la carte.
Reynolds *et al.* 2000 [48]	28 elementary schools in Alabama (USA) assessed the effects of a dietary intervention program in 4th graders over two years based on diet and psychosocial variables.	The High 5 project included classroom, parent, and cafeteria intervention components that increased availability of F&V in alliance with education.	F&V consumption was significantly higher in the intervention group children at follow-up one and two when compared to children in the control group. F&V consumption by parents in the intervention group was significantly higher at follow-up one when compared to control parents.
Osganian *et al.* 1996 [49]	The CATCH Eat Smart Program was implemented at 56 schools in four states over 2.5 years and assessed school menu, recipe, and vendor product information on five consecutive days on three occasions.	Targeted school food service staff through education on making positive changes in the nutrient quality of school meals and base them on national dietary goals to lower the total fat, saturated fat, and sodium content of school meals.	There was a significantly greater mean reduction in the calories from total fat and saturated fat in intervention compared with control school from baseline to follow-up. Though total caloric consumption decreased by 683kcal n the intervention group it was still one-third above the Recommended Dietary Allowances for this age group.
Bartholomew & Jowers. 2006 [50]	Two elementary schools of similar size and demographic data in Texas (USA) were used for a two-phase study evaluating an intervention to increase selection of low- and moderate- fat entrees over two semesters.	In Phase 1, the rotation of existing entrees was modified such that one of three entree choices was low or moderate in fat. In Phase 2, the number of competing high-fat entrees was reduced from two choices to one.	In Phase 1 in the intervention school, the number of days that a low-fat entree was offered increased by 70%, with no increase in the rate of selection of the lower moderate-fat entrees. In Phase 2, both low- and moderate-fat entrees were selected at a significantly higher rate in the intervention school (32.1% and 26.4%, respectively) than the control school (13.8% and 7.5%, respectively).
Belansky *et al.* 2010 [51]	The project surveyed 45 low-income, rural elementary schools in Colorado one year before and after a healthy eating, wellness policy mandated in 2006.	The What’s Working project described the influence of a mandated Local Wellness Policy (LWP) to identify impacts, opportunities, and barriers.	Three improvements were associated with the new policy, namely: increased percentage of schools with policies stipulating healthy items be offered in classroom parties (21.4% in 2005 *vs.* 48.7% in 2007), daily fresh fruit be offered in lunchrooms (0.80 choices in 2005 *vs.* 1.15 choices in 2007), and skinless poultry be used (27% in 2005 *vs.* 59% in 2007).
Anderson *et al.* 2005 [52]	Investigated the impact of a school-wide nutrition education program in primary schools in Scotland at baseline and 9 months. Dietary and attitude assessments of children aged 6–7 and 10–11 were performed.	Increased provision of F&V and provided point-of-purchase marketing materials, education materials, newsletters, and teacher information.	Children in the two intervention schools had a significantly higher average increase in fruit consumption than those in two control schools. No other changes in nutrient uptake were detected.

### 3.2. Interventions in Worksites

A summary of effective strategies to increase healthy food intake in the workplace has been developed previously [53,54], and six articles on healthy food procurement in worksites were included in this review. A study at several worksites in Denmark incorporated education with healthy food procurement strategies and provided greater access to fruits and vegetables and found increased consumption of healthy foods by 70 grams per day [55]. Similarly, increasing the availability of healthy foods and educating staff about the importance of a healthy diet was an effective means of improving healthy food intake by up to 20% among staff at multiple worksites (Table 2) [56,57,58]. Two worksite interventions reduced the availability of unhealthy nutrients in workplace foods (e.g., energy from fat reduced by 30% and sodium by up to 65% per serving) while increasing healthier food options in a cafeteria and vending machines (Table 2) [41,59]. Reducing relative pricing on low-fat snacks was effective in increasing low-fat snack purchases from vending machines in adult and adolescent populations (Table 2) [41]. Further, when available and properly marketed, customers may accept healthy food options over unhealthy alternatives (Table 2) [41,59]. 

**Table 2:** Healthy Food Procurement Programs in Worksites

Study and Year	Study Description	Intervention(s)	Post-intervention Outcome(s) and Notable Findings
Lassen *et al.* 2004 [55]	Five worksites in Denmark with canteens promoted healthier lunches with an end point and follow up data collection.	Implemented a continuous quality improvement of canteen lunches through a spectrum of strategies to include increased availability of healthy foods, staff training, goal setting, and support groups.	On average across the five sites there was 70g /day/customer increase in the intake of F&V intake at endpoint and a 95 g/day/customer increase four months after endpoint.
Beresford *et al.* 2001 [56]	Targeted 28 Seattle, WA (USA) worksites with cafeterias in Seattle to increase F&V intake assessed at baseline and two-year follow-up.	Seattle “5 a Day for Better Health” is a simple message encouraging people to eat more F&V which was launched at 14 intervention worksites and compared with 14 control worksites.	Significantly higher intake of F&V in the intervention group after two years with 0.3 more servings than the control group.
Sorensen *et al.* 1999 [57]	22 Community Health Centers in Massachusetts (USA) implemented the Treatwell 5-a-day project to get participants to consume >five F&V servings per day.	The program incorporated three interventions, namely minimal intervention, worksite intervention, and worksite plus family intervention which included education components.	Total intake increased by 19% in worksite plus family group, 7% in worksite group, and 0% in minimal intervention group. Only 23% of all participants reported consuming more than five servings per day. Consumption of F&V was directly associated with level of household support for healthy eating.
Sorensen *et al.* 1998 [58]	24 manufacturing worksites in Massachusetts (USA) assessed the impact of an integrated health promotion effort.	Implemented three intervention components: joint worker-management participation in program planning and implementation, consultation with management on worksite environment, and health education.	The intervention group had a reduced intake of calories consumed as fat (2.3% *vs.* 1.5% in control) and increased intake of F&V (10% *vs.* 4% in control.
French *et al.* 2001 [41] (Also in Table 1)	Examined the impact of pricing and promotion of low-fat snacks in vending machines at 12 worksites and 12 in Minnesota (USA).	Low-fat snacks added to 55 vending machines were subject to four pricing conditions and three promotional conditions. Sales and profits were tracked over a 12-month period.	Price reductions of 10%, 25%, and 50% were associated with significant increases in sales of low-fat snacks in adults and adolescents. Profits per vending machine were not impacted and promotional signage may have had slight effect.
Perlmutter *et al.* 1997 [59]	Assessed acceptance of more healthful entrees in a Kansas (USA) worksite cafeteria that services est. 200 employees per day based on sales data, nutrient analysis, customer acceptability.	Five phase study modified entrees over a 7-month period to include less than 30% energy from fat and less than 1,000 mg sodium per serving. A marketing component identified healthier food offerings.	No significant difference in sales was observed though customers may be more willing to accept changes in flavor attributes when they are identified as healthful and include nutrient information.

### 3.3. Interventions in Hospitals, Care Homes, Correctional Facilities, Government Institutions and Miscellaneous Settings

Outside of school and worksite settings, hospitals, care homes, correctional facilities, government institution, and a few miscellaneous settings have implemented healthy food policies and programs (Table 3). In Ireland, the impact of a structured catering initiative on food choices was evaluated in a hospital setting [60]. A cross-sectional comparison was made using a 24-hour dietary recall and questionnaire of participants aged 18–64 years in two hospitals; one implemented a catering initiative that promoted nutritious food and reduced sugar, fat, and salt, and the other was used as a control (Table 3) [60]. Overall, this study found that improving the dietary quality of menu items provided in hospitals can reduce the amount of unhealthy nutrients such as fat, sugar, and sodium in foods served to patients in a hospital setting by up to 30% [60]. In England, the Food Standards Agency introduced healthy nutrition standards, to include reduced fat and increased fruit and vegetable intake, for persons >75 years of age in residential and nursing care homes though outcomes in these settings have not been reported upon [61]. Yet, homebound, low-income seniors that were delivered healthy food baskets increased their intake of fruits and vegetables relative to a control group (Table 3) [62]. In addition, interventions have been introduced in some correctional facilities. For example, the Indiana Department of Correction (IDOC) and their food-service provider (ARAMARK Correctional Services) collaborated to create a new menu that substantially improved the dietary quality of foods in all 28 facilities across the state of Indiana in the United States (Table 3) [27].

**Table 3:** Healthy Food Procurement Programs in Hospitals, Care Homes, Correctional Facilities, Government Institutions, and Miscellaneous Settings.

Study and Year	Study Description	Intervention(s)	Post-intervention Outcome(s) and Notable Findings
L’Abbé *et al.* 2011 [26]	Comprehensive review on existing healthy food procurement policies and programs.	Details multiple programs and their interventions on healthy food procurement initiatives.	Numerous successful food procurement programs in Canada and Internationally are described to include criteria (such as sodium limits) for healthy foods and recommendations for a healthy food procurement framework in Canada.
CDC 2012 [27]	28 correctional facilities across Indiana (USA).	Implemented new menu with 20% less sodium than the previous diet.	Successfully launched healthier food menu statewide. Menu also increased servings of fruit in place of baked desserts, averaging at least five servings of F&V per day. To help lower cholesterol, the menu also serves no fried foods and fewer high-fat menu items.
Geaney *et al.* 2011 [60]	Two public hospitals in Ireland and monitored food and nutrient intake monitored for participants aged 18–64 in their canteen.	One of the two hospitals implemented a catering initiative designed to provide nutritious foods while reducing sugar, fat, and salt intakes in their canteen.	Mean intakes of total sugars, total fat, saturated fat, and salt were significantly lower in the intervention hospital where 72% of respondents, compared with 42% in the control, complied with the recommended under-3 daily servings of food high in fat and sugar. In the intervention hospital, 43% of respondents exceeded the recommended salt intake of 4–6 g/day, compared with 57% in the control.
Johnson *et al.* 2004 [62]	480 homebound, low-income seniors receiving Meals on Wheels over 4 months in Seattle, WA (USA)	Increased access to fresh F&V via home delivery.	Seniors receiving baskets consumed 1.04 more servings than those in the control group. The number of seniors consuming >five servings per day increased by 17% from baseline.
Vander Wekken & Naylor 2010 [63]	48 local governments in British Columbia, Canada, including 12 First Nations addressed food environments in 142 community funded facilities.	Evaluated food and beverage framework in local recreational settings during 2008–2010.	The initiative was successful at facilitating changes in policy, practices, food provision, and patron awareness. Key factors for change and challenges to implementation were identified.
PSFPI 2009 [64]	Comprehensive initiative for food public institutions such as schools, hospitals, and prisons in the United Kingdom.	Developed and disseminated the PSFPI report to encourage consumption of locally grown foods and availability of healthy foods and build momentum for progress.	Awareness of the program increased by 24% in 2 years; 72% of local authorities and 69% of schools supported initiative; 54% of users find the guidelines very useful or extremely useful; constraints were identified.

Notes: CDC: Centers for Disease Control and Prevention; USA: United States of America; F&V: fruit and vegetables; FSA: Food Standards Agency; PSFPI: Public Sector Food Procurement Initiative.

In February 2010, Alberta Health Services (AHS) introduced detailed dietary guidelines for AHS facilities for planning menus that meet nutritional targets from each food group and also nutrient criteria, such as the amount of sodium in a standard item [26]. The guidelines were divided into foods “recommended” and “not recommended” which included recommended servings per day of each category. For example, sodium levels in foods such as soups, frozen vegetables, yogurt, chocolate and soy milk, cookies, crackers, pancakes, waffles, cereal bars, and cheese were addressed across the province [26]. An evaluation in August 2010 found that the revised menu met the sodium target of <3,000 mg/day which is still higher than the dietary guidelines set in Canada [26]. The province continues to monitor the nutrient content of the menu and target comparisons twice per year. Similarly, British Columbia, Canada introduced healthy food policies in all recreational facilities and government buildings across the province, to include 12 First Nations, with successful impact [63]. Their healthy food policy interventions have led to 91% of vending machine food offerings being healthy compared to 35% prior to the intervention [63]. Meanwhile, community gardens in six California communities increased the consumption of fruits and vegetables as well as physical activity of participants (Table 3) [65].

In the United Kingdom, the 2002 Curry Report provided 100 recommendations designed to revive the role of farmed foods with consumers while achieving a more competitive and sustainable food supply [26]. Similarly, the “Public Sector Food Procurement Initiative (PSFPI)” was updated in 2011 by the Department of Environment, Food, and Rural Affairs to encourage the public sector to work with farmers to ensure that sustainable, healthy, and nutritious food is consumed in a variety of venues such as schools, hospitals, and correctional facilities [26,27]. Effective, best practices and barriers to food procurement were identified and guides and toolkits were developed to aid the broad implementation of healthy food procurement strategies (Table 3) [26,65]. In Norway, the price of foods (subsidies, taxes based on food nutritional quality) was found to be the primary method of influencing healthy choices [66]. Further, reducing the price of healthy foods such as grain, low fat milk, and vegetables and increasing prices for unhealthy foods such as sugar and butter was speculated to improve health outcomes [66].

### 3.4. Interventions in Remote Communities

The *Healthy Foods North* (HFN) program was a multilevel health intervention program aimed at improving the diet and nutritional status in six Inuit communities in the Canadian Arctic [67]. Specifically, the HFN intervention increased the availability of affordable/healthy foods (traditional foods, fruits, vegetables, and low sugar beverages), decrease the availability of less healthy foods and beverages (low in nutrients, high in fats and sugars), and promoted physical activity [67]. The HFN decreased intake of total calories and carbohydrate and average BMI by 2.6% [67]. Another healthy food intervention implemented in remote communities in Northern Canada is the Food Mail Project program [68]. This program aimed to reduce the cost of healthy perishable foods, increase nutrition education, and promote healthy foods in retail settings as a means to improve nutrition and health in the isolated communities [68]. An analysis of household surveys indicated that there was an increase in the purchase of fresh/frozen fruits and vegetables, milk, and eggs across all communities, and, in some cases, there was also an increase in the sale of other foods such as cheese and yogurt [68]. Both the HFN and Food Mail Project demonstrated that increased access to and consumption of quality, healthy food is achievable in remote communities where there are considerable logistical challenges though behavior change occurred slowly [67,68]. In 2005, a “Retail Based Nutrition Intervention” promoted healthier grocery store environments in Northern, isolated First Nations and Inuit communities in Canada [69]. By improving the availability and affordability of 32 targeted healthy foods while disseminating educational resources, the program found an initial increase in healthy food sales but that positive impact was not maintained after the promotion activities ended [69].

### 3.5. Discussion

Where evaluated, healthy food procurement programs found in this review were nearly always effective at increasing availability of healthier food and decreasing that of less healthy food; contributing to the increased purchases of healthier foods and lower purchases of food high in fat, sodium and sugar. Further, some interventions that included a health parameter as an outcome, found that healthy food uptake led to improvements in health outcomes (blood pressure and BMI) [39,42]. Although poorly documented in most studies, some interventions were “popular”, some improved attitudes towards healthy eating, and some observed increases in total food sales as well as that of healthier foods. Health economic modeling from Los Angeles suggested that an effectively and broadly implemented government healthy food procurement policy could reduce disease rates and health costs while one of the interventions noted substantive cost advantages [36,70]. Our review has found evidence supporting the effectiveness of healthy food procurement policies at increasing healthy eating in a variety of settings.

There are, however, multiple limitations to the positive conclusions of this review. There were limited interventions in remote communities and no interventions found in low and middle income countries (LMIC). Most of the studies in this review were from the UK, Canada and USA and were limited to settings where the populations are relatively ‘captive’ with very few interventions in community or commercial settings. It is possible that in ‘free living’ situations (e.g., outside public institutions such as schools or hospitals) people will simply purchase food elsewhere. In the evaluated studies, additional health or policy interventions were often included with healthy food procurement interventions. These ancillary interventions often included educational programs (in schools, through public workshops, and online programs), price reductions or subsidies for healthy foods, and in one study, a physical activity program was included [38]. These interventions seemed to increase the impact of the food procurement policy and may be important success factors. It was not possible to assess the impact of food procurement separately from the ancillary interventions. 

Another limitation to this review was the difficulty in locating studies evaluating food procurement policies. These policies are often implemented by governments with the outcomes potentially not being published, (even when indicated they are being assessed) or published in less accessible “grey” literature. It is likely that our search for policy evaluations missed several studies. The authors tried to mitigate this likelihood by directly contacting multiple experts including those in government and the WHO. Similarly, it is possible that the restricted nature of the search terms used in databases excluded studies that could have been included in the review. Lastly, we cannot exclude that there is a publication bias in the studies we identified. 

We did not find any unsuccessful policy interventions. However, the Canadian media in 2012 released a story of an organized student protest relating to a provincial government health food procurement policy. Gum, coffee, chocolate, French fries, soda, pizza, and other foods were removed from schools, which has resulted in opposition from students who protested for the re-introduction of these foods, arguing that the policy has removed their freedom of choice [71]. The applicability of healthy food procurement policies to communities and in commercial settings, the barriers and challenges to the policies, long term impact on food purchases and consumption, costs of the intervention, sustainability, need for and usefulness of ancillary healthy eating policies (e.g., education and costing of food), and the utility of food procurement policy intervention in LMIC represent some future policy research needs. Increased priority funding from national funding organizations to support research on how to improve healthy eating such as healthy food procurement policies are needed. Such studies could include large scale randomized controlled trials with health outcomes and economic analysis as critical outcomes. 

Healthy food procurement policies may be implemented for a variety of reasons in addition to having a direct impact on food purchases. Healthy food procurement policies have been indicated to increase the capacity of the food industry to produce healthy foods or to reformulate product lines to be healthier. This may only be a factor for policy interventions that affect large populations (e.g., national or regional government, large employer or bulk food procurer such as a major grocery store chain). Our review did not find any evaluations of the impact of policy on food manufacturers. Apart from the impact on health outcomes, in many countries food procurement is implemented to strengthen the local agriculture industry and or to reduce the overall costs of food purchases and the health impact is secondary. These latter purposes were not evaluated in this analysis but represent potential, additional rationale for introducing a healthy food procurement policy. It is also recommended that healthy food procurement policies are made necessary for schools, employers and governments to be internally consistent with the stated public policies relating to the health of those who consume the food they procure. Governments almost universally advocate healthy eating, schools teach students about healthy eating, and are in part responsible for students’ wellbeing, while hospitals have responsibility for improving the health of those they care for and employers often have policy and priorities for creating healthy, safe workplaces. Procuring unhealthy food especially for relatively captive populations in these settings may be inconsistent with stated goals, priorities or other policies and has potential to undermine the credibility of the procuring organization. 

## 4. Conclusions

Although many research questions remain about healthy food procurement policies, our review directly supports implementation of such policy in schools, worksites, and government institutions. Additional settings where people have limited eating options (hospitals, care homes, correctional facilities, military bases, and remote communities) would also likely to be able to introduce policy and successfully impact healthy eating. In the absence of contradictory evidence or rationale, we recommend broadly implementing (and evaluating) healthy food procurement policy for all settings where food is purchased by government or non-government organizations. Prior or simultaneous implementation of ancillary education about healthy eating and supportive pricing policy are likely to be critical success factors. Several documents have been developed to aid and encourage the uptake of healthy food procurement policies in different settings [24,27,28,31,54].
